# Development and psychometric evaluation of a 360-degree evaluation instrument to assess medical students’ performance in clinical settings at the emergency medicine department in Iran: a methodological study

**DOI:** 10.3352/jeehp.2024.21.7

**Published:** 2024-04-01

**Authors:** Golnaz Azami, Sanaz Aazami, Boshra Ebrahimy, Payam Emami

**Affiliations:** 1 Department of Emergency Medical Sciences, Faculty of Paramedical Sciences, Kurdistan University of Medical Sciences, Sanandaj, Iran; 2 Department of Nursing and Midwifery, Faculty of Nursing and Midwifery, Ilam University of Medical Science, Ilam, Iran; Hallym University, Korea

**Keywords:** Emergency medical services, Iran, Leadership, Psychometrics, Statistical factor analysis

## Abstract

**Background:**

In the Iranian context, no 360-degree evaluation tool has been developed to assess the performance of prehospital medical emergency students in clinical settings. This article describes the development of a 360-degree evaluation tool and presents its first psychometric evaluation.

**Methods:**

There were 2 steps in this study: step 1 involved developing the instrument (i.e., generating the items) and step 2 constituted the psychometric evaluation of the instrument. We performed exploratory and confirmatory factor analyses and also evaluated the instrument’s face, content, and convergent validity and reliability.

**Results:**

The instrument contains 55 items across 6 domains, including leadership, management, and teamwork (19 items), consciousness and responsiveness (14 items), clinical and interpersonal communication skills (8 items), integrity (7 items), knowledge and accountability (4 items), and loyalty and transparency (3 items). The instrument was confirmed to be a valid measure, as the 6 domains had eigenvalues over Kaiser’s criterion of 1 and in combination explained 60.1% of the variance (Bartlett’s test of sphericity [1,485]=19,867.99, P<0.01). Furthermore, this study provided evidence for the instrument’s convergent validity and internal consistency (α=0.98), suggesting its suitability for assessing student performance.

**Conclusion:**

We found good evidence for the validity and reliability of the instrument. Our instrument can be used to make future evaluations of student performance in the clinical setting more structured, transparent, informative, and comparable.

## Graphical abstract

[Fig f2-jeehp-21-07]

## Introduction

### Background/rationale

The 360-degree evaluation method provides students with multiple pieces of feedback that can help them evaluate and improve their performance from various perspectives. Furthermore, the 360-degree evaluation process can provide teachers/medical experts with a deeper insight into students’ clinical competency and performance, while improving student and staff satisfaction with the evaluation process [1].

While 360-degree assessments offer valuable insights, few 360-degree assessment tools are tailored for medical students, hindering their ability to receive feedback from various sources. This mirrors the challenges faced in healthcare settings, where multisource feedback, despite its benefits, remains hampered by tool limitations. Several barriers obstruct the widespread use of 360-degree assessments in healthcare settings. First, most feedback tools are not designed specifically for healthcare administrators, but rather for managers in other industries. Second, cost is a major hurdle. Finally, developing accurate and reliable 360-degree assessments in-house presents a challenge for many healthcare organizations. The specialized knowledge required to design and deliver effective surveys can be a significant barrier. This suggests a need for readily available, high-quality tools specifically tailored for the healthcare administration context [2].

### Objectives

This study aimed to develop and validate a 360-degree tool for medical experts to comprehensively assess prehospital emergency medical service students’ clinical performance.

## Methods

### Ethics statement

Ethical approval was obtained from the Kurdistan University of Medical Sciences Ethics Committee for Research Involving Human Subjects (IR.MUK.REC.1402.029). Written and oral informed consent was obtained from each participant before enrollment. Students were informed that participation was voluntary and anonymous, that all answers would be treated as confidential, and that non-participation would have no influence on their university standing or grades.

### Study design

Both qualitative and quantitative approaches were used to develop and validate the 360-degree evaluation instrument.

### Setting

This study was conducted at the Department of Emergency Medical Sciences at the Faculty of Paramedical Sciences, Kurdistan University of Medical Sciences, in Sanandaj, Iran. The study targeted second and third-year undergraduate students enrolled in the prehospital emergency medical service program. These students were actively engaged in clinical placements at the time of invitation, ensuring a practical context for their participation. The methods of scale development, determining the content validity, and determining the face validity are described in detail in Supplement 1.

### Measurement tool (instrument)

In Supplement 1, the initial number of items were 58. It was reduced to 55 after two rounds of Delphi evaluation and adjustment. The full version of the instrument consisted of 55 items divided into 6 subscales: leadership, management, and teamwork (19 items), consciousness and responsiveness (14 items), clinical and interpersonal communication skills (8 items), integrity (7 items), knowledge and accountability (4 items), and loyalty and transparency (3 items). The instrument used a 4-point Likert scale for ratings (to avoid the middle tendency of the test samples and force a specific response), with 1=rarely (<25%), 2=sometimes (25%–50%), 3=often (50%–75%), and 4=almost always (>75%). Boxes with the option “not applicable” were added for all individual items, in case the participant had no experience with the individual item. The instrument scores are calculated as sums of item scores, with higher scores indicative of better performance. When scoring, the “not applicable” option should be regarded as a missing value according to the scoring manual. Items are rated such that higher scores indicate better performance. The subscale scores are computed as the sum of the items divided by the number of items answered. If “not applicable” is marked for any of the items, then their total score for the subscale is divided by the number of items the respondent actually answered, rather than the number of the items in the subscale. If more than half of the items in a subscale are missing, the subscale score should not be computed [3].

To maximize accessibility and impact, the instrument, initially developed in Persian, underwent a rigorous translation process following the World Health Organization guidelines for backward-forward translation to ensure accuracy and cultural appropriateness for English speakers, broadening its potential user base [4]. The present study examined the psychometric properties of the Persian version of the 360-degree evaluation instrument in a sample of prehospital emergency medical service students. Our study instrument is presented in Supplement 2 in English and Persian.

### Pilot testing

In order to test our developed instrument in a wider context, a pilot study with a cross-sectional design was conducted from May 2023 to July 2023. A purposive sample of undergraduate prehospital emergency medical service students who are currently registered at the department were invited to take part in this study. Potential participants were contacted by phone to set up an appointment to have the instrument administered at an office in the faculty. Those who attended received an exploratory statement outlining the purpose of the study beforehand and gave their informed consent. Two hundred and ninety-five individuals participated in the pilot testing of the instrument.

### Bias

The pilot study used purposive sampling, which may introduce selection bias. This method targets a specific group and may not be representative of the wider population the instrument is intended for.

### Study size

The sample size for the pilot study was based on Hair’s criteria (2010) for acceptable factor analysis, which recommends the inclusion of at least 5 participants per question [5]. Since our instrument had 55 items, 300 participants were required to be recruited, considering a drop-out rate of 10%.

### Statistical analysis

Two software packages IBM SPSS and AMOS ver. 25.0 (IBM Corp.) were used for statistical analysis. All P-values presented were 2-sided, and the significance level was set at 0.05. Exploratory factor analysis (EFA) and confirmatory factor analysis (CFA) were used to examine the psychometric properties of our instrument. EFA was conducted using the principal component extraction method with varimax rotation [6].

Subsequently, CFA was conducted using AMOS (IBM Corp.), and several indices were applied to assess the model fit. The model fit was examined using the goodness of fit index (GFI) with an acceptable level of >0.90, root mean square error of approximation (RMSEA) with an acceptable level between 0.05 and 0.08. comparative fit index (CFI) with an acceptable level of >0.90, and normal fit index (NFI) with acceptable level of >0.90. The convergent validity of the instrument was also evaluated [7]. The average variance extracted (AVE) was computed. Cronbach’s α was calculated to determine the internal consistency of the instrument. Test-retest reliability was used to assess the instrument’s consistency and reliability.

## Results

### Demographic characteristics of the study participants

In total, 300 students were eligible to participate in this study. Of the invited students, 2 agreed to participate in the study but did not attend, 1 did not respond (unsuccessful phone call after 6 repeated attempts), and 2 stated that they were not interested after reading the information. The remaining 295 participated in the study. Nearly 19% of the respondents were women (N=55). On average, participants completed the instrument in 20 minutes. Table 1 presents the demographic characteristics of the study participants.

### Main results

The raw response data are available in Dataset 1.

#### Content validity

The content validity of the instrument was checked through a consultation with experts. Agreement among the experts was high (item CVI=0.93, scale CVI/avg=0.87) and all the items on the tool were considered necessary by the experts (content validity ratio=0.95).

#### Face validity

The instrument’s face validity was measured using the FVI, item FVI, and scale FVI values. The FVI values range from 0 to 1, with an FVI of at least 0.83 considered acceptable. The item FVI ranged from 0.83 to 1, and the scale FVI for the total score was 0.91, suggesting that the instrument has excellent face validity. The instrument items were slightly modified based on the expert feedback to fit our population of interest.

#### Construct validity testing

EFA was conducted using the principal component extraction method with orthogonal rotation (varimax). The Kaiser-Meyer-Olkin (KMO) measure verified the sampling adequacy of the analysis (KMO=0.96; “superb” [6]), and all KMO values for individual items were >0.40, demonstrating sufficient fit of the instrument items for factor analysis. Bartlett’s test of sphericity yielded results of χ^2^ (1,485)=19,867.99, P<0.01, indicating that correlations between items were sufficiently large for principal component extraction. An initial analysis was run to obtain eigenvalues for each component in the data. Six components had eigenvalues over Kaiser’s criterion of 1 and in combination explained 60.1% of the variance. The scree plot was slightly ambiguous and showed inflections that would justify retaining both components 2 and 4. Given the adequate sample size, the convergence of the scree plot, and the satisfaction of Kaiser’s criterion for 6 components, 6 components were retained in the final analysis. Supplement 3 shows the factor loading after rotation, presenting the items that clustered on the same components.

**Fig. 1.**
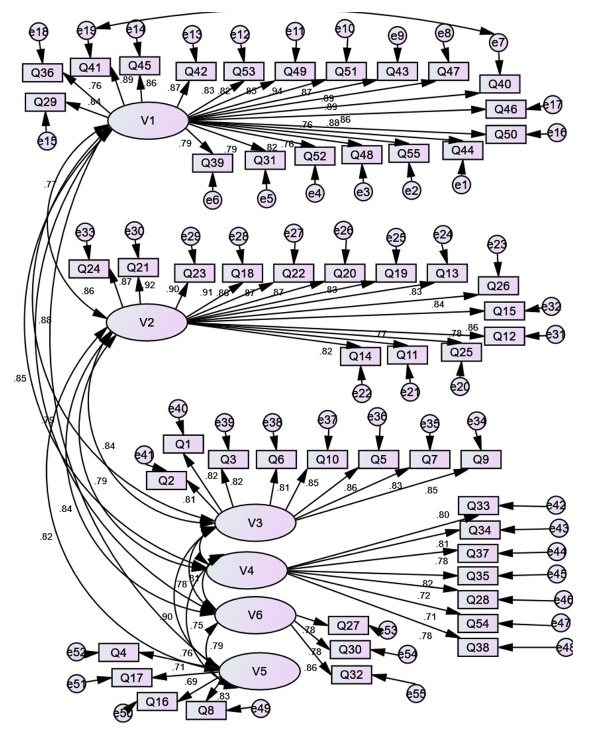
Confirmatory factor analysis of the 360-degree evaluation instrument.

The 1-factor model extracted from EFA was examined in AMOS ver. 21.0 (IBM Corp.) using the maximum likelihood estimation method. The results of CFA to test the construct with 6 dimensions yielded a chi-square of 599.81 (degrees of freedom=41, P=0.000) (Fig. 1).

The GFI was created as an alternative to the chi-square test, and it determines the proportion of variance that is accounted for by the estimated population covariance. The GFI in this study was 0.91, showing a good fit for the unidimensional model of the instrument construct. Furthermore, the results of CFA in the current study were as follows: RMSEA=0.06, CFI=0.93, NFI=0.91, and TLI=0.90. All of the reported indices imply good fit for this model with the 55-item 360-degree evaluation instrument construct.

#### Discriminant validity

Discriminant validity was evaluated to ensure that each latent variable in the model can be discriminated from the other 5 constructs. We calculated the AVE for each latent variable using the formula presented in Table 2. When the AVE for each latent variable is greater than the shared variance of the other variables, discriminant validity is established. The AVEs and shared variances are presented in Table 2. Adequate evidence was found to support the 6-dimensionality of the 360-degree evaluation instrument.

#### Convergent validity

Convergent validity can be established if the AVE for all dimensions is greater than 0.50. Our analysis showed that the AVE values for the 6 dimensions of the 360-degree evaluation instrument were greater than 0.50, confirming the convergent validity.

#### Internal consistency analysis

Internal consistency reliability was evaluated using Cronbach’s α. Cronbach’s α coefficient was 0.98 for the total instrument (55 items) in the final version, which was considered acceptable. The internal consistency for the 6 subscales of the instrument was as follows: leadership, management, and teamwork (α=0.97), consciousness and responsiveness (α=0.97), clinical and interpersonal communication skills (α=0.94), integrity (7 items, α=0.91), knowledge and accountability (α=0.83), and loyalty and transparency (α=0.84).

#### Test-retest reliability

Test-retest reliability was assessed by asking a subgroup of our participants (N=30) after a 2-week period to complete a second copy of the instrument. The stability (test-retest) reliability of the instrument was determined using Spearman correlation coefficients. The test-retest correlation coefficient for the total scale was 0.90 (P<0.05). The correlation coefficients between the test and retest for each subscale of the instrument ranged from 0.81 to 0.93. Thus, the results indicate that the total scale and its subscales had adequate stability and reliability over time.

## Discussion

### Key results

The authors developed an instrument that initially contained 58 items in 6 dimensions, from which 55 items were retained after conducting the content validity analysis. The study findings indicate that the 360-degree evaluation instrument is a valid and consistent instrument, with excellent internal consistency and adequate stability and reliability, that can be used to assess students’ performance.

### Interpretation

From a conceptual point of view, this is the first instrument designed to assess the performance of prehospital emergency medical service students on their clinical placement that incorporates multiple perspectives. The 360-degree evaluation method assesses the student’s clinical competence, interpersonal skills, communication abilities, and degree of professionalism, integrating feedback from multiple sources. The 360-degree feedback approach has been used extensively in the fields of business and aviation. Interestingly, this approach has recently been introduced into medical education. Multisource feedback has clearly become such an integrated part of the assessment process for students in Iran that the absence of a validated 360-degree evaluation tool in the prehospital emergency medical service field must be justified. This instrument was developed and validated by a panel of experts with a strong background in nursing education, making it appropriate for use in nursing contexts.

In this study, we adopted an evidence-based multi-perspective approach to develop and validate the instrument. The content of our developed instrument was driven by the relevant literature, existing guidelines, students’ perspectives, and professional experts. These procedures—in combination with the theoretical constructs embodied by the instrument and expert review of the items—helped confirm its face and content validity. In the future, more studies need to be conducted to further clarify the acceptance of our developed instrument, both from the specialist’s and the student’s perspectives.

### Comparison with previous studies

The 360-degree evaluation method is a new paradigm in medical education in which trainees are assessed by multiple people in their sphere of influence [8]. During supervised clinical training, students are expected to develop a range of professional competencies essential to providing safe, high-quality care [9]. Since 1993, 360-degree evaluation, or multisource feedback, has been a widespread practice in professional competency assessment [10]. The 360-degree assessment involves external evaluation of students’ performance by (1) a peers, (2) the healthcare team (nurses, allied healthcare professionals), (3) the trainee’s self-evaluation, and (4) patients or their relatives. The 360-degree assessment may help to uncover blind spots, which are clinical behaviors that the student is unaware that he/she does or does not do and can consist of unknown weaknesses or hidden strengths [1]. Globally, hospitals and medical education boards are increasingly expanding their trainee evaluation and review approach to prioritize patient safety, with a greater focus on accountability and professionalism [1].

### Limitations

Our instrument was developed and validated using only a sample of university students in Iran. More studies are needed to assess the applicability of this instrument in different populations or other culturally specific settings in order to confirm its field performance. The cross-sectional design of this study is yet another limitation, since the study findings may not be generalizable to a population with diverse ethnic and educational backgrounds. In this study, we did not assess convergent and divergent validity. Moreover, we did not calculate the multi-rate kappa, which may provide additional evidence for the content validity of the instrument. However, the findings regarding both item CVI and scale CVI, in combination with the comments from the panelists, sufficed to validate the content of our instrument.

### Generalizability

This instrument offers educators and curriculum planners a potential tool for evaluating emergency medical service students’ clinical performance. The generalizability of the study findings is limited by its specific context and population, but the potential benefits extend beyond the original setting and can inform future research and practice advancements in prehospital emergency medical service education globally.

### Conclusion

The researchers built and tested a new tool to assess the clinical performance of prehospital emergency medical students. This tool uses feedback from multiple sources (360-degree feedback) and was validated through expert review and a pilot study. The new tool offers a clearer, more informative way to evaluate student performance in real-world settings.

## Figures and Tables

**Figure f2-jeehp-21-07:**
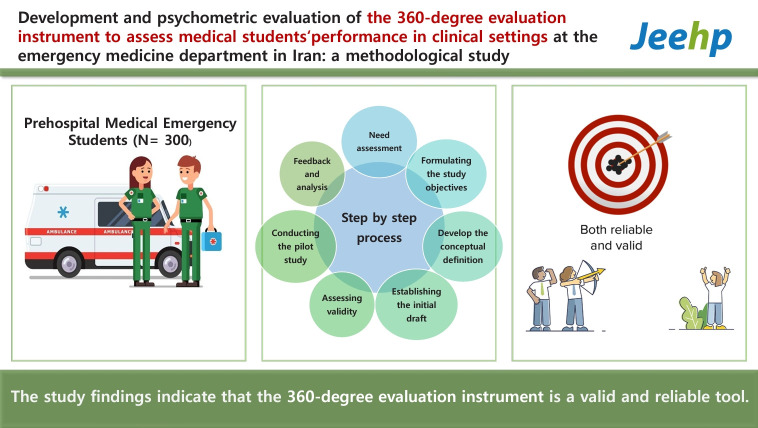


**Table 1.** Demographic characteristics of the participants

Variable	Category	Frequency (%)
Gender	Men	240 (81.3)
	Women	55 (18.7)
Age (yr)	20–25	295 (100.0)

Total number of participants=295.

**Table 2.** Shared variance (square of correlation) and discriminant validity

	AVE	V1	V2	V3	V4	V5	V6
V1	0.72	1	0.71^**^	0.54^**^	0.60^**^	0.53^**^	0.64^**^
V2	0.73	0.71^**^	1	0.65^**^	0.56^**^	0.56^**^	0.56^**^
V3	0.69	0.54^**^	0.65^**^	1	0.42^**^	0.56^**^	0.48^**^
V4	0.61	0.60^**^	0.56^**^	0.42^**^	1	0.43^**^	0.50^**^
V5	0.57	0.53^**^	0.56^**^	0.56^**^	0.43^**^	1	0.42^**^
V6	0.66	0.64^**^	0.56^**^	0.48^**^	0.50^**^	0.42^**^	1

$\mathrm{AVE}=\frac{\sum_{i=1}^{n}\;\lambda^{2}}{n}$; *λ*=standardized factor loading, *n*=number of items. AVE, average variance extracted.

** P<0.01 (Correlations are significant at P<0.01).

## Footnotes

**Data availability**
Data files are available from Harvard Dataverse: https://doi.org/10.7910/DVN/VU213G Dataset 1. Raw response data from students to the measurement tool to assess the performance of prehospital medical emergency students in Iran.

## Supplementary materials

Supplementary files are available from Harvard Dataverse: https://doi.org/10.7910/DVN/VU213G

Supplement 1. Methods of scale development, determining the content validity, and determining the face validity.

Supplement 2. The 360-evaluation instrument to assess the performance of prehospital medical emergency students.

Supplement 3. The 6 factors, with their loading items, of the 360-evaluation instrument to assess the performance of prehospital medical emergency students.

Supplement 4. Audio recording of the abstract.
