# Supplementary material for: Development and psychometric evaluation of a 360-degree evaluation instrument to assess medical students’ performance in clinical settings at the emergency medicine department in Iran: a methodological study
Source: J Educ Eval Health Prof. 2024 Apr 1;21:7. doi: 10.3352/jeehp.2024.21.7 (PMC11078574; doi:10.3352/jeehp.2024.21.7)
Supplement: Supplementary file 2 — Supplement 1. Methods of scale development, determining the content validity, and determining the face validity. [file jeehp-21-07-suppl1.docx]

Supplement 1. Methods of scale development, determining the content validity, and determining the face validity

Scale development

To develop a relevant and effective survey, we first needed to clearly define the intended audience as well as scope and domain of its content. For our purpose, the intended audience is emergency medical service students. The scope and domain are clinical performance of students. The definition of "medical emergency students' clinical performance" can vary depending on the specific context and program. However, generally, it refers to the demonstration of knowledge, skills, and behaviors expected of a student pursuing a career in emergency medicine while providing patient care in a clinical setting. Here are some key aspects that might be included in this definition:

**Knowledge:**

- Understanding of emergency medical protocols and procedures
- Ability to diagnose and manage common medical emergencies
- Knowledge of pharmacology and medications used in emergency settings
- Staying up-to-date with the latest advancements in emergency medicine

**Skills:**

- Performing physical examinations and assessments
- Administering medications and other interventions
- Operating medical equipment
- Communication and teamwork skills in high-pressure situations
- Critical thinking and problem-solving

**Behaviors:**

- Professionalism and ethical conduct
- Empathy and compassion towards patients
- Effective communication with patients and healthcare team members
- Time management and ability to prioritize tasks effectively
- Adaptability and resilience in stressful situations

To ensure our 360-degree feedback tool comprehensively assessed emergency medical service students' clinical performance, we took a multi-step approach. We started by searching on different databases, without a time limit, with the keywords “36-degree feedback”, “Student performance appraisal”, “clinical education”, “tools”, “psychometric”, and “medical emergency students”. While tools existed for nursing, residents, and medical students, none focused on emergency medical service students. By reviewing existing literature, we pinpointed areas where current tools are lacking and identified specific elements that could benefit our study. To ensure the developed assessment tool adhered to ethical principles and professional standards specific to the Iranian context, the study additionally reviewed relevant code of ethics and professional conduct guidelines [1] published in Iran. Then, to define the exact scope and range of " emergency medical service students' clinical performance," we analyzed their approved curriculum and extracted a detailed blueprint outlining all the expected competencies and skills they need to demonstrate in the clinical setting. To gain further insights and perspectives, we conducted individual interviews with five faculty members, whose expertise directly informed the development of our assessment tool.

At this stage researchers identified 6 factors to be included in the first draft of our newly developed tool: (1) leadership, management, and teamwork; (2) consciousness and responsiveness; (3) Clinical and interpersonal communication skills; (4) integrity; (5) knowledge and accountability; (6) loyalty and transparency. The initial exploration yielded 58 relevant items. The items of the newly developed tool were initially assessed using the Delphi methodology [2]. Three faculty members were asked to evaluate the quality of generated items and eliminate those provided to be inadequate. The items on the instrument were modified based on the expert`s feedback. After two rounds of Delphi evaluation and adjustment, 55 items remained on the initial version of the instrument. The instrument was also given to 10 students (other than the subjects of this study). As a result, the instrument items were modified to clarify the meaning by using easier words or clarifying explanations was added. Item development was followed by a pre-feasibility test, which allowed us to provide valuable feedback to modify and improve the instrument items.

## Determining the content validity

In this phase, the researchers established the content validity of the developed instrument Content validity is concerned with the extent to which the scope or range of items is used to measure the variable [3]. A panel expert of 5 members including a nursing educator (N= 4) and emergency medical educator (N= 1) examined the initial instrument. The panel was asked to comment on each item regarding accuracy, clarity, simplicity, ambiguity, and style. Based on the panelist feedback, items were slightly modified to fit the purpose of the study. Subsequently, a different panel of 6 experts on nursing education (N=4), emergency medical education (N=1), and health education (N=1) were asked to comment independently on the relevance and necessity of the instrument items in order to calculate the content validity index (CVI) and content validity Ratio (CVR) respectively [4].

The validity of each instrument domain was evaluated using the CVI. The relevance of the items was assessed using a four-point Likert scale (1= not relevant; 2 slightly relevant; 3 relevant’ 4 very relevant). Rates reported by the panel of experts were used to calculate the CVI. The panelist rating of 1 and 2 represent irrelevant or invalid content and ratings of 3 and 4 represent relevance and valid content. The evaluation sheet also provided space next to each item where the experts could provide comments or suggestions. The content validity of the instrument was examined by measuring the CVI in three indexes, including the item level CVI (I-CVI), scale level CVI (S-CVI), and CVR for each item`s relevancy [4].

To calculate the I-CVI the rating of either 3 or 4 on the four-point scale was divided by the total number of experts. The I-CVI values range from 0 to 1, with the I-CVI of ≥ 0.79 indicating the highest level of item relevancy, 0.70 to 0.79 indicates the item needs revision, and ICVI< 0.70 suggests that the lower level of relevancy (items need to be eliminated) [4]. In line with widely acceptable recommendations for assessing I-CVI where more than 5 expert raters, a mean value of 0.78 for I-CVI was set to determine the acceptable value [4].

The S-CVI was calculated using two methods: (1) universal agreement (S-CVI/UA), and (2) average or mean export proportion (S-CVI/avg). To calculate the S-CVI/UA the rating of either 3 or 4 on the four-point scale was divided by the total number of items. The S-CVI/avg was calculated by summing the average of the I-CVI scores of all items and dividing them by the total number of items. The S-CVI/UA is sensitive to the number of experts. The S-CVI/UA tends to decrease when greater than 2 experts are involved. The SCVI-avg is more liable and recommended by Polit and Beck [5]. In general, an S-CVI rating of ≥ 0.80 is considered acceptable [4]. The preliminary version of the instrument was fine-tuned by the research team based on the values achieved by the items for I-CVI and S-CVI.

The necessity of the instrument items was assessed to determine the CVR of our developed instrument. CVR is a method for measuring mutual agreement among judges or rates regarding how essential a particular item is [6]. The CVR was calculated based on the responses of the panelists rating each item as (1) essential, (2) useful, but not essential, and (3) not necessary [6]. For each item, the total number of raters who chose “essential” was calculated. The following formula was used to calculate the CVR:

$$CVR= \frac{n_{e}-( \frac{N}{2})}{\frac{N}{2}}$$

Where $n_{e}$ = the number of panelists indicating “essential”, and N total number of panelists. CVR value ranges from -1 to +1, where a value inclining toward +1 indicates the mutual agreement of the experts on the respective item. On the contrary, a negative CVR value may be obtained when less than half of the panelists choose the item as “essential” [6]. In general, higher scores reflect the greater agreement among the panelists on the item`s necessity. A CVR rating of at least 0.78 is considered acceptable, otherwise, the individual item should be either revised or deleted [7]. The panel of experts was asked to make 55 sets of ratings where each set serves as an instrument item.

## Determining the Face Validity

Next, face validity was performed in order to provide the pre-final version of the instrument. An instrument is considered to have face validity when it appears to a group of experts to be able to measure what it is supposed to measure [3]. The face validity of the instrument was assessed by an expert panel of 5 specialists in emergency medical sciences, and nursing sciences, to ensure that the wording was correct and the meaning of each sentence was easily understandable for the participant. The panelist was asked to rate each item of the instrument regarding the clarity and comprehensibility of each item. The questionnaire rating was on the four-interval scale (1= Neither clear nor understandable; 2 somewhat clear and understandable; 3= clear and understandable; 4 very clear and understandable), with ratings of 1 and 2 representing irrelevant or invalid content and ratings of 3 and 4 represent valid and relevant content [8]. After that, the face validity of the instrument was tested by measuring the face validity index (FVI), including item-level FVI (I-FVI) and scale-level FVI (S-FVI) [8]. To calculate the I-FVI the rating of either 3 or 4 on the four-point scale (agreed item) was divided by the number of experts. The S-FVI (average calculation) was calculated by averaging the I-FVI scores for all items across all experts. Then, the provisional instrument was then administered to 10 prehospital emergency medical service students in order to examine the clarity and readability of the items. All participants stated that they had no problem in reading and understanding the items.

**References**

1. Shamsi-Gooshki E, Parsapoor A, Asghari F, Parsa M, Saeedinejad Y, Biroudian S, Fadavi M, Khalajzadeh MR, Namazi HR, Ghasemzadeh N. Developing" code of ethics for medical professionals, medical council of Islamic Republic of Iran". Arch Iran Med 2020;23:658-664. <https://doi.org/10.34172/aim.2020.83>

2. Nasa P, Jain R, Juneja D. Delphi methodology in healthcare research: how to decide its appropriateness. World J Methodol 2021;11:116. <https://doi.org/10.5662/wjm.v11.i4.116>

3. Nieswiadomy RM, Bailey C. Foundations of nursing research. 7th ed. Sydney: Pearson Education; 2018. p. 26-75.

4. Souza ACd, Alexandre NMC, Guirardello EdB. Psychometric properties in instruments evaluation of reliability and validity. Epidemiol Serv Saúde 2017;26:649-659. <https://doi.org/> <https://doi.org/10.5123/S1679-49742017000300022>

5. Polit DF, Beck CT. The content validity index: are you sure you know what's being reported? Critique and recommendations. Res Nurs Health 2006;29:489-497. <https://doi.org/> <https://doi.org/10.1002/nur.20147>

6. Binti Hassan SA. Content validity of STEMTIP using CVR method. Int J Acad Res Bus Soc Sci 2018;8:1118-1125. <https://doi.org/10.6007/IJARBSS/v8-i7/4559>

7. Murrar S, Brauer M. The SAGE encyclopedia of educational research, measurement, and evaluation: Mixed model analysis of variance: SAGE Publications; 2018. p. 1075-1078.

8. Yusoff MSB. ABC of response process validation and face validity index calculation. Educ Med J 2019;11. <https://doi.org/> <https://doi.org/10.21315/eimj2019.11.3.6>
