# Supplementary material for: Development and psychometric evaluation of a 360-degree evaluation instrument to assess medical students’ performance in clinical settings at the emergency medicine department in Iran: a methodological study
Source: J Educ Eval Health Prof. 2024 Apr 1;21:7. doi: 10.3352/jeehp.2024.21.7 (PMC11078574; doi:10.3352/jeehp.2024.21.7)
Supplement: Supplementary file 4 — Supplement 3. The 6 factors, with their loading items, of the 360-evaluation instrument to assess the performance of prehospital medical emergency students. [file jeehp-21-07-suppl3.docx]

| **Supplement 3.** The six factors with their loading items | | | | | | |  |
| --- | --- | --- | --- | --- | --- | --- | --- |
| **6^th^ Factor** | **5^th^ Factor** | **4^th^ Factor** | **3^rd^ Factor** | **2^nd^ Factor** | **1^st^ Factor** | **Items** |  |
|  |  |  |  |  | 0.80 | در برخورد با بيماران به تمام ابعاد جسمي، رواني واجتماعي آنان توجه می کند  The student pay attention to patients as an individual and is concern for the physical, psychological, and social aspects of their disease. | **1** |
|  |  |  |  |  | 0.74 | در صورت نیاز، با سایر اعضای تیم ارائه دهنده ی خدمات سلامت همکاری به موقع و مناسبی دارد تا طرح درمانی یکپارچه و مستمری برای بیمار ارائه دهد  To provide an integrated and dynamic treatment plan for the patient, the student worked in collaboration with other members of the healthcare providers as needed. | **2** |
|  |  |  |  |  | 0.72 | در تمامي مراحل مراقبت از بيماران وقت كافي صرف می کند  The student spends enough time in all stages of the patient care process | **3** |
|  |  |  |  |  | 0.71 | مستندات مربوط به وضعیت بیمار یا مراقبت های انجام شده برای او را به طور کامل و دقیق ثبت می کند  The students provide a complete and accurate record of the patient`s condition and the medical care given to the patient | **4** |
|  |  |  |  |  | 0.70 | در برخورد با بیمارو سایر اعضای تیم مراقبتی رفتار حامي و عادلانه دارد  The student has a fair and supportive behavior when interacting with patients and other healthcare professionals | **5** |
|  |  |  |  |  | 0.69 | توانايي در مديريت موثر زمان و تعيين اولويت ها در مراقبت از بیمار را دارد  The student has the ability to manage time effectively and set priorities for patient care | **6** |
|  |  |  |  |  | 0.69 | به خواسته ها و آلام بيماران توجه می کند  The student can advocate for their patient's needs and has a reasonably high regard for each patient's wishes | **7** |
|  |  |  |  |  | 0.68 | به جایگاه دیگران احترام می گذارد و به صورت سازنده و مثبت در گروه کار می کند  The student is a great team member. He/she respects the position of others and works constructively and positively in the group | **8** |
|  |  |  |  |  | 0.67 | در مواجهه با بیماران مختلف عدالت را بدون توجه به نژاد، مذهب، عقیده، جنسیت و ... رعایت می کند  The student observes justice in dealing with different patients regardless of race, religion, opinion, gender, etc. | **9** |
|  |  |  |  |  | 0.67 | به همکاران احترام می گذارد و در صورت نیاز با آن ها ارتباط موثر برقرار کرده و اطلاعات لازم را فراهم می کند  As a team member, the student respects his/her colleagues communicates with them effectively, and provides invaluable feedback as needed | **10** |
|  |  |  |  |  | 0.66 | توانايي تشخيص و ارزش گذاشتن به نقش ديگران را دارد  The student has the ability to recognize and value the role of others | **11** |
|  |  |  |  |  | 0.66 | منشور حقوق بيمار را در شرايط مختلف رعايت كرده و از نقض نشدن حقوق بیمار اطمینان حاصل می کند  Respect the patient's right charter under different conditions and ensure that the patient's rights are not violated | **12** |
|  |  |  |  |  | 0.58 | کلیه موارد پذیرش، بستری، یا اختلالات ایجاد شده در روند درمان بیمار را به اطلاع مربی، پرستاران/یا پرسنل بخش می رساند  All cases of patient admission, hospitalization, or difficulties in the treatment process are reported to the trainer, Nurses/or hospital ward staff | **13** |
|  |  |  |  |  | 0.54 | قضاوت یا پيش داوری نمي كند  The student does not judge or prejudice | **14** |
|  |  |  |  |  | 0.53 | بازخورد کلامی و کتبی سازنده را به سایر اعضای تیم مراقبتی بهداشتی ارائه می دهد  The student provides constructive verbal and written feedback to other members of the healthcare team | **15** |
|  |  |  |  |  | 0.51 | در صورت مشاهده ی خطای پزشکی همکار، موضوع را بدون قضاوت با وی مطرح می سازد تا فورا اقدامات اصلاحی یا پیشگیرانه انجام شود  If the student notices a medical error from a colleague, approach the colleague and gently bring up the situation in a non-judgmental way to take immediate corrective or preventive measures | **16** |
|  |  |  |  |  | 0.50 | منافع و مصالح بیمار را محور تصمیمات پزشکی می داند و آن را بر امور و مناقع شخصی خود ترجیح می دهد  The student ensures that the patient's best interest is considered at the center of medical decisions, regardless of the student`s personal opinion | **17** |
|  |  |  |  |  | 0.48 | رازداری را درباره ی اطلاعات بیمار رعایت می کند  Maintain patient confidentiality | **18** |
|  |  |  |  |  | 0.41 | فعالانه به دنبال مشاوره، کمک یا راهنمایی از اساتید، معلمان و سایر دانش جویان است تا دانش و مهارت های فعلی خود به روز کند  The student actively seeks advice, assistance, or direction from professors, teachers, and other students in order to keep up-to-date with current knowledge and skills | **19** |
|  |  |  |  | 0.74 |  | به بیمار به عنوان یک انسان منحصر به فرد رفتار کنید و به فردیت بیمار احترام بگذارید  Treat each patient as a unique being and have respect for the patient's individuality | **20** |
|  |  |  |  | 0.73 |  | با بیمار و همراهان وی با عزت و احترام برخورد می کند  Treat the patients and their companions with dignity and respect | **21** |
|  |  |  |  | 0.71 |  | به عقايد، آداب ، رسوم وعادات بيمار احترام بگذارند.  Respect the patient's beliefs, culture, customs, and practice | **22** |
|  |  |  |  | 0.69 |  | در صورت مرخصي رفتن یا غیبت کردن ، هماهنگي هاي لازم را انجام مي دهد  In case of going on leave of absence or being absent, the student makes the necessary arrangement | **23** |
|  |  |  |  | 0.68 |  | حریم خصوصی بیمار را رعایت می کند  Respect patient privacy | **24** |
|  |  |  |  | 0.66 |  | قوانین و مقررات گروه و بیمارستان را رعایت می کند  Follow the rules and regulations of the department and hospital | **25** |
|  |  |  |  | 0.65 |  | آراستگی ظاهری و پوشش حرفه ای مناسب دانشجو بالینی را مطابق با خط مشی محیط بالینی حفظ می کند  The student maintains a professional appearance and dress code in accordance with the policy of the clinical setting | **26** |
|  |  |  |  | 0.65 |  | همیشه در ساعات موظف کار در بیمارستان نشان شناسایی اش را در محل قابل دید لباس خود نصب می کند  Wear the identification badge in a visible position at all times during working hours in the hospital | **27** |
|  |  |  |  | 0.61 |  | نسبت به اساتید، پیشکسوتان، و افراد با سابقه تر از خود با احترام و حق شناسی رفتار می کند  Treat professors, seniors, and people with more experience with respect and dignity | **28** |
|  |  |  |  | 0.59 |  | در سریع ترین زمان ممکن، رفتارهای مراقبتی را با بکارگیری حداکثر تلاش برای پاسخگویی به نیازها و درخواست های بیمار انجام می دهد  The students' caring attention is accomplished by applying maximum efforts to respond to the patient's needs and requests as quickly as possible | **29** |
|  |  |  |  | 0.57 |  | وظایف خود را مسئولانه و با بالاترین استانداردهای ایمنی انجام دهند  Perform her/his duties responsibly and to the highest safety standards | **30** |
|  |  |  |  | 0.56 |  | ادب و اخلاق حرفه ای به ویژه فروتنی و برخورد مناسب با اساتید، کارکنان، بیماران و ... را رعایت می کند  Demonstrate professional courtesy and respect when communicating with professors, colleagues, patients, etc. | **31** |
|  |  |  |  | 0.55 |  | وقت شناس است و به موقع در بخش حاضر می شود  He/she is always punctual and shows up to work on time | **32** |
|  |  |  |  | 0.53 |  | مسئولیت پذیر و  پاسخگو در ارائه مراقبت به بیمار است  Demonstrates responsibility and accountability for the care provided | **33** |
|  |  |  | 0.78 |  |  | 1. معاینات بالینی را بر اساس روش های استاندارد انجام می دهد.   She/he performs the clinical examinations according to standardized methods. | **34** |
|  |  |  | 0.78 |  |  | 1. گرفتن شرح حال از بیمار را به صورت دقیق و کامل انجام می دهد و اطلاعات دقیق و معتبری را ارائه می دهد   The student provides accurate and authoritative information by taking a careful and complete history | **35** |
|  |  |  | 0.76 |  |  | 1. در حین گرفتن شرح حال، انجام معاینه بالینی، و یا آموزش به بیمار یا خانواده وی از مهارت های برقراری ارتباط به درستی استفاده می کند   Uses appropriate communication skills while taking a history, performing a clinical examination, or teaching the patient or family | **36** |
|  |  |  | 0.64 |  |  | 1. پروسیجرهای مراقبتی و درمانی که بر عهده وی قرار داده شده با حوصله، به موقع و به درستی انجام می دهد (از قبیل تعبیه لوله معده (NG Tube) ، گذاشتن سوند ادراری، خونگیری شریانی (ABG)، رگ گیری محیطی، خون گیری و ...)   She/he patiently, timely, and correctly performs the care and treatment procedure (such as nasogastric tube (NG tube, urinary catheterization, arterial blood gas (ABG), Insertion of a Peripheral Intravenous Cannula, blood collection, etc.) | **37** |
|  |  |  | 0.60 |  |  | 1. استفاده درست و صحیح از وسایل و تجهیزات بخش و نحوه ی مراقبت و نگهداری از آن ها را می داند   She/he knows the correct use of the medical equipment and the necessity of regular maintenance of the equipment and checks for safety | **38** |
|  |  |  | 0.59 |  |  | 1. از وضعیت بیمار خود (ارزیابی، تشخیص، و درمان) به خوبی آگاه است   She/he is well aware of the patient's situation (assessment, diagnosis, and treatment plan) | **39** |
|  |  |  | 0.57 |  |  | 1. ارائه آموزش دقیق، با کیفیت بالا و دلسوزانه به بیمار به گونه ای که برای بیمار و اعضای خانواده او قابل درک باشد.   Deliver accurate, high-quality, and compassionate patient education in a way that is understandable by the patients and their family members | **40** |
|  |  |  | 0.52 |  |  | 1. دانش کافی در مورد نحوه تشخیص و درمان اختلالات رایج در بالین را دارد   She/he has adequate knowledge of how to diagnose and treat common disorders in clinical placements | **41** |
|  |  | 0.79 |  |  |  | انتقاد پذير است  She/he is open to criticism | **42** |
|  |  | 0.77 |  |  |  | در بحث با همکار، او ظرفیت بالایی برای پذیرش استدلال منطقی نشان می دهد و برای حفظ فضای محترمانه و سازنده در بحث تلاش می کند (پرهیز از پرخاشگری، انتقاد مخرب و بی اساس).  In discussion with a colleague, she/he shows a great capacity to accept logical reasoning and strive to maintain a respectful and constructive atmosphere in the discussion (avoid aggressiveness, destructive, and baseless criticism) | **43** |
|  |  | 0.63 |  |  |  | از هرگونه مشاجره لفظی و برخورد فیزیکی اجتناب می کند  She/he avoids all confrontation, both verbal and physical | **44** |
|  |  | 0.59 |  |  |  | به دنبال دریافت بازخورد از عملکرد خویش است و وقتی به وی بازخورد داده می شود می پذیرد  She/he seeks to receive feedback on her performance and accepts it when it is given | **45** |
|  |  | 0.55 |  |  |  | او همیشه در شرایط سخت آرام و خونسرد است. خونسردی خود را حفظ می کند و توانایی حل سریع و کارآمد مشکلات را دارد  she/he is always calm and composed in difficult situations. She/he keeps cool and can solve problems quickly and efficiently | **46** |
|  |  | 0.48 |  |  |  | در بالین، کلیه اقدامات درمانی و تشخیصی تحت نظارت کامل متخصصان واجد الشرایط انجام می دهند  At clinical placement, all diagnostic measures and treatments are performed under the full supervision of qualified professionals | **47** |
|  |  | 0.44 |  |  |  | محدوديت هاي علمي خودرا شناخته ، در موارد لازم مشاوره و كمك می خواهد  She/he knows her/his scientific limitations and asks for advice and help if necessary. | **48** |
|  | 0.63 |  |  |  |  | برای کسب آمادگی لازم علمی در موضوعات درسی مربوطه به میزان کافی مطالعه می کند  To get scientifically prepared, she/he studies relevant subjects to a sufficient extent | **49** |
|  | 0.59 |  |  |  |  | به منظور مقابله با سو رفتار حرفه ای همکارانش، موارد مشکوک به سو رفتار حرفه ای یا بی کفایتی در همکارانش را گزارش می دهد  Report suspicions of impairment or incompetence concerning their colleagues to tackle professional misconduct | **50** |
|  | 0.57 |  |  |  |  | روند مراقبت از بیماران را پیگیری می نماید و از ارائه اطلاعات لازم برای ادامه ی مراقبت از آن ها پس از ترخیص اطمینان حاصل می کند  She/he Follows up on the outcomes of all patients cared for and ensures the provision of the necessary information for patients continued after the discharge | **51** |
|  | 0.57 |  |  |  |  | در بحث های گروهی و کنفرانس های آموزشی مشارکت فعال دارد  She/he actively participate in presentation of academic conferences and group discussions | **52** |
| 0.65 |  |  |  |  |  | در صورت حضور بیماران و همراهان، از تصمیمات سایر همکاران انتقاد نمی کند و صرفا در صورت درخواست بیمار یا تردید بالینی راه های قانونی پیگیری پیش بینی می شود (بدون قضاوت کردن عملکرد همکاران)  In the presence of patients and companions, she/he does not criticize the colleagues` decisions, and follow-up interventions are anticipated only at the patient`s request or due to clinical doubt (without judging the performance of her/his colleague) | **53** |
| 0.54 |  |  |  |  |  | در صورت بروز خطای پزشکی، ضمن پذیرش مسئولیت، خطای خود را به پزشک/استاد گزارش می دهد تا اقدامات اصلاحی هر چه سریع تر انجام شوند  In the event of a medical error, while accepting the responsibility, she/he takes immediate corrective measures by informing the patient`s doctor/professor of the mistake so that the action can be made as soon as possible | **54** |
| 0.50 |  |  |  |  |  | اگر از توانایی و مهارت لازم برای انجام مراقبت و برآورده کردن نیازهای بیمار برخوردار نیست، از اساتید یا سایر دانشجویان کمک می گیرد و بیمار را نیز از این موضوع آگاه می سازد  If she/he is not competent enough to meet the patient's needs and provide care with high quality, ask for help from the professors or peers and inform the patient about it. | **55** |
